# New insights into targeted therapy of glioblastoma using smart nanoparticles

**DOI:** 10.1186/s12935-024-03331-3

**Published:** 2024-05-07

**Authors:** Habib Ghaznavi, Reza Afzalipour, Samideh Khoei, Saman Sargazi, Sakine Shirvalilou, Roghayeh Sheervalilou

**Affiliations:** 1 Pharmacology Research Center, Zahedan University of Medical Sciences, Zahedan, Iran; 2 Molecular Medicine Research Center, Hormozgan Health Institute, Hormozgan University of Medical Sciences, Bandar Abbas, Iran; 3 Department of Radiology, Faculty of Para-Medicine, Hormozgan University of Medical Sciences, Bandar Abbas, Iran; 4 Finetech in Medicine Research Center, Department of Medical Physics, School of Medicine, Iran University of Medical Sciences, Tehran, Iran; 5 Cellular and Molecular Research Center, Research Institute of Cellular and Molecular Sciences in Infectious Diseases, Zahedan University of Medical Sciences, Zahedan, Iran; 6 Department of Clinical Biochemistry, School of Medicine, Zahedan University of Medical Sciences, Zahedan, Iran

**Keywords:** Smart nanoparticles, Glioblastoma (GBM), Therapy, Clinical trials

## Abstract

In recent times, the intersection of nanotechnology and biomedical research has given rise to nanobiomedicine, a captivating realm that holds immense promise for revolutionizing diagnostic and therapeutic approaches in the field of cancer. This innovative fusion of biology, medicine, and nanotechnology aims to create diagnostic and therapeutic agents with enhanced safety and efficacy, particularly in the realm of theranostics for various malignancies. Diverse inorganic, organic, and hybrid organic–inorganic nanoparticles, each possessing unique properties, have been introduced into this domain. This review seeks to highlight the latest strides in targeted glioblastoma therapy by focusing on the application of inorganic smart nanoparticles. Beyond exploring the general role of nanotechnology in medical applications, this review delves into groundbreaking strategies for glioblastoma treatment, showcasing the potential of smart nanoparticles through in vitro studies, in vivo investigations, and ongoing clinical trials.

## Introduction

### Glioblastoma (GBM): epidemiology, subtypes

There are two types of brain tumors based on the place of origination: primary and metastatic. While the latter is self-explanatory, primary brain tumors are those that originate from within the cranium [1]. Glioma is a term that denotes brain tumors originating from cells of glial phenotype. This category of cancerous growths encompasses astrocytoma, ependymoma, oligoastrocytoma, oligodendroglioma and several less common variants with atypical histopathologic features. In the field of medicine, gliomas constitute approximately 25% of all primary brain masses [2]. The most up-to-date WHO guidelines for classifying neoplastic masses of the CNS adopt markers of genetic and epigenetic nature. However, gliomas can also be categorized based on a grading system; namely low-grade gliomas (LGG) and high-grade gliomas (HGG) which correspond to WHO grades 1–2 and 3–4, respectively [3].

According to the WHO CNS5 classification, CNS tumors are dividing to 6 different groups including:

1) Adult-type diffuse gliomas (the majority of primary brain tumors, including (a) Astrocytoma, isocitrate dehydrogenase (IDH)-mutant, (b) Oligodendroglioma, IDH-mutant and 1p/19q-codeleted, (c) glioblastoma multiforme (GBM), IDH-wild type)

2) Pediatric-type diffuse low-grade gliomas (with good prognoses)

3) Pediatric-type diffuse high-grade gliomas (aggressive type)

4) Circumscribed astrocytic gliomas (with solid growth pattern)

5) Glioneuronal and neuronal tumors (with neuronal differentiation)

and 6) Ependymomas [4].

The prevailing glioma is GBM, essentially an astrocytoma of WHO grade 4, which represents 14.3% of primary CNS neoplasms, constituting 49.1% of all malignant brain tumors [2, 3]. GBM as a strongly aggressive neoplasm of the CNS [5, 6] correlated with very unfavorable patient prognosis [7] which the 5-year survival is roughly 7% [2]. The average survival for affected people is less than two years, 15 months after diagnosis, making GBM responsible for 4% of cancer-related deaths [5, 6, 8, 9]. According to Siminska et al. in 2021, the incidence of GBM varies in different populations, and is reported to be 3.20 (Ostrom et al. in 2017) [10], 4.06 (Walker et al. in 2019) [11], 4.17 (Fabbro-peray et al. in 2019) [12], 4.40 (Gittlemao et al. in 2018) [13] and 4.64 (Brodbelt et al. in 2019) [14] per 100,000 people by different investigations. Therefore, GBM is a rare disease [15, 16], according to whose guidelines the disease is categorized into four major types based on histopathological features [17].

In order to enhance our understanding of the molecular foundations of GBM, comprehensive analyses have been conducted [3]. In 2010, these analyses facilitated the categorization of glioblastomas into subtypes based on their transcriptional activity. These subtypes include classical (characterized by EGFR+, CDKN2A−, and an absence of TP53 mutations), mesenchymal (featuring altered NF1, PTEN mutations, heightened transcription of CD44, MET and MERTK), proneural (exhibiting altered PDGFRA, mutated TP53, IDH1 point mutations, and OLIG2 upregulation), and neural (expressing GABRA1, NEFL, SYT1, and SLC12A5) [18]. Median survival for the mesenchymal, classical, and proneural subtypes are 11.5, 14.7, and 17.0 months, respectively [19]. Diagnosing GBM relies significantly on three key molecular changes: the simultaneous gain and loss of chromosomes 7 and 10 (+ 7/-10), TERT promoter mutation, and EGFR amplification. Such molecular markers aid in identifying the tumor as GBM, even when histological examination might imply a low-grade tumor [20].

### Routine theranostic challenges

#### Diagnostics

The established method for radiographic characterization of GBM is magnetic resonance imaging (MRI), widely utilized for diagnosis and post-therapeutic management [21]. Additionally, to identify risk factors tests based on computed tomography (CT) or MRI, fundamental tools for glioma detection, should be incorporated [22].

The current WHO classification is notably intricate and advocates for an integrated diagnosis, considering both histopathological and molecular typing that incorporates genetic mutations and molecular markers [17, 23]. A conclusive diagnosis relies on histopathological examinations of the tumor or its parts, obtained intraoperatively, using conventional histological, cytological, and histochemical methods. In cases where neurosurgical lesion excision is not feasible, a fine-needle aspiration (FNA) biopsy is recommended [24, 25]. Glial fibrillary acidic protein (GFAP), part of the cytoskeletal protein family, is extensively expressed in astroglial and GBM cells. The absence of GFAP expression indicates significantly undifferentiated tumor cells but does not suggest tumor progression. Therefore, serum GFAP can be considered a candidate biomarker in diagnosing GBM [26].

#### Therapies

Standard therapy for malignant gliomas involves the administration of chemotherapy, radiotherapy, and interventional surgical procedures [27]. Surgery followed by temozolomide-based chemoradiotehrapy is the standard treatment in the setting of early diagnosis. There is no standard of care in the case of relapse, but, according to the patient’s conditions, radiotherapy, surgery, and systemic treatment with chemotherapy or bevacizumab might be indicated [8, 28]. Nevertheless, the outcomes concerning survival extension and treatment response are inconsistent with both chemotherapy and radiotherapy [29].

#### Challenges

In spite of progress in diagnosing and treating GBM, the prognosis, incidence, and mortality rates continue to be unfavorable [30].

MRI serves as the prevailing standard for diagnosing and monitoring newly identified and recurrent masses. The outcomes derived from MRI are crucial for pre-treatment characterization and assessing the response to therapy. However, challenges arise as conventional MRI faces difficulty distinguishing between primary tumors and metastases, as well as CNS masses, and determining true progression versus pseudoprogression. Radiological features of these conditions often overlap. Gliomas, metastatic lesions, and primary CNS lymphomas typically manifest as contrast-enhancing tumors surrounded by T2-hyperintense edema [31]. Furthermore, glioblastomas exhibit similarities in metabolite ratios, making cutting-edge imaging techniques insufficient. Another obstacle in diagnosis of glioma is the substantial intertumoral heterogeneity, challenging the idea that gliomas originate from a single cell [32]. Differential patterns of copy number alteration (CNA) have been suggested to influence tumor development [17].

A significant hurdle in glioblastoma treatment is tumor recurrence, with a median survival of 14.6 months for GBM patients undergoing conventional multimodal therapies. The progression-free survival (PFS) for recurrent GBM does not reach 24 weeks. Drawbacks in conventional GBM treatment, such as neurotoxic effects and unsatisfactory loading efficiency limit its therapeutic potential [30].

Additionally, the blood-brain barrier (BBB) plays a central role in restricting therapeutic strategies, as numerous drugs exhibit little or no solubility to cross this physical barrier [30]. The BBB, a specialized system in the brain’s vasculature, regulates molecular transport across the endothelial wall [33]. In many regions of the CNS, the vasculature comprises endothelial cells linked by tight junctions, situated alongside pericyte on a basement membrane. These cells, alongside neighboring neurons and microglia, facilitate cerebral immune responses, primarily aiming to safeguard this intricate organ [34]. The transportation of most molecules is orchestrated by different receptors within the BBB [35]. It is not uncommon for the BBB to undergo impairment due to specific pathologies, such as neoplasms. Brain masses can disrupt the normal functioning of the BBB [33], due to the positive regulatory effect they exert on angiogenesis [36].

Temozolomide (TMZ), which is known as the gold standard of treatment, promotes tumor progression and angiogenesis by alteration of IL8/CXCL2/CXCR2 signaling. Urbantat et al. investigated modifications in the signaling pathway during the recurrence of human glioblastoma multiforme and explored the specific impact of TMZ. They also established a combination therapy involving TMZ and CXCR2 antagonization to evaluate its effectiveness and tolerability. The study revealed a significant reduction in the infiltration of tumor-associated microglia (TAM), with high TAM infiltration in primary tumors correlating with reduced overall survival (OS). Moreover, more patients exhibited IL8 expression, and TMZ therapy maintained the expression of CXCL2. In rodents, the combination therapy demonstrated enhanced anti-tumoral effects. Additionally, the combinatorial therapy confers promise for overcoming CXCR2-mediated resistance [37]. Although focal radiotherapy increases the average survival, it may cause cognitive impairment, DNA damage and other severe systemic side effects. In studies, the anti-angiogenic drug bevacizumab was found to result in prolonged PFS when administrated in combination with chemotherapy for newly diagnosed GBM, albeit, the non-significant effect on OS as observed in phase III trials [38]. Chinot et al. assessed the impact of incorporating bevacizumab into the radiotherapy–temozolomide regimen in glioblastoma treatment. The inclusion of bevacizumab alongside radiotherapy–temozolomide was not accompanied by enhanced survival for glioblastoma patients. However, bevacizumab was associated with more frequent adverse events compared to the placebo [39]. Lack of specificity, unwanted cytotoxicity and multi-drug resistance (MDR) stand among the most impactful challenges associated with the current chemotherapeutic regimens [40]. Generally, Surgery, radiotherapy, and chemotherapy as existing treatments for GBM ultimately increase the patient’s survival by only a few months [7].

### New approaches in glioblastoma control

The limitations of the effectiveness of existing therapies for GBM and the existence of mechanisms that contribute to therapeutic resistance emphasize the necessity for the creation of innovative diagnostic strategies [41]. Considering the constraints of current diagnostics and therapeutics, there is a significant need for developing novel diagnostics and therapeutics for glioblastoma. For these novel theranostics to be successful, they must be specific, controllable, capable of crossing the BBB, and demonstrate efficacy. A range of new nanomedicines has emerged to address this gap. Additionally, leveraging computational methods and bioinformatics databases could enhance the management of glioblastoma [42].

#### Computational methods and bioinformatic databases

Various computational models have been created to capture different facets of glioblastoma, and these simulation tools can be employed to forecast tumor expansion, evaluate the impact of disrupting molecular pathways in specific brain regions, and comprehend the considerable heterogeneity within the tumor microenvironment [43, 44]. In general, these models can be categorized into distinct groups, ranging from simplified models that simulate only tumor volume growth to intricately detailed models that encompass numerous genetic or proteomic processes implicated in the development and progression of glioblastoma [45]. As researchers gain a deeper understanding of the biological complexity, modeling strategies have progressed to offer insights into glioblastoma across various scales (tissue, cellular, and molecular) [46] encompassing essential glioma behaviors like vascularization, diffusion, and invasion capacity [47]. Improved modeling contributes to more effective therapy optimization tailored to each cancer patient [42]. Molecular signaling pathway analysis aids in quantitatively identifying potential cancer targets, and mathematical models of mass transport phenomena enable predictions regarding drug delivery to the brain, assisting in experimental design [48].

#### Bioinformatic analysis

Numerous bioinformatic studies have been carried out to uncover new targets for regulating GBM progression. Leveraging public genomic databases like TCGA, REMBRANDT, Gravendeel, KEGG, and CGGA, researchers have sought to identify therapeutic targets and construct predictive nomograms [49]. Ongoing investigations aim to discover novel biomarkers for identifying molecular pathways [50], employ advanced genome editing technologies such as CRISPR-Cas or CRISPR-Cas9 to overcome chemotherapeutic resistance, utilize targeted miRNAs to silence genes promoting autophagy, and explore the use of plant-derived bioflavonoids to inhibit autophagy and enhance the therapeutic efficacy of TMZ in GBM [41].

#### Nanotechnology

The field of nanotechnology has brought about a revolution in the conventional methods of treating, diagnosing, and managing gliomas. This transformation is largely attributed to late progresses in bioengineering, improved drug accessibility, and the ability to specifically target cancer cells by accumulating and entrapping them [30]. Nanomaterials (NMs), such as metal and polymer-based nanoparticles [51] are being increasingly used in the field of cancer theranostics [52], owing to their small size, large surface area, specific structural features, binding affinity, cell membrane or tissue penetration capability, and long elimination half-life in the circulation [53–55]. The high surface-to-volume ratio of nanoparticles enables them to deliver small biomolecules such as nucleic acids, proteins and drugs to the target site and increase the efficacy of therapeutic agents [53].

**Fig. 1:**
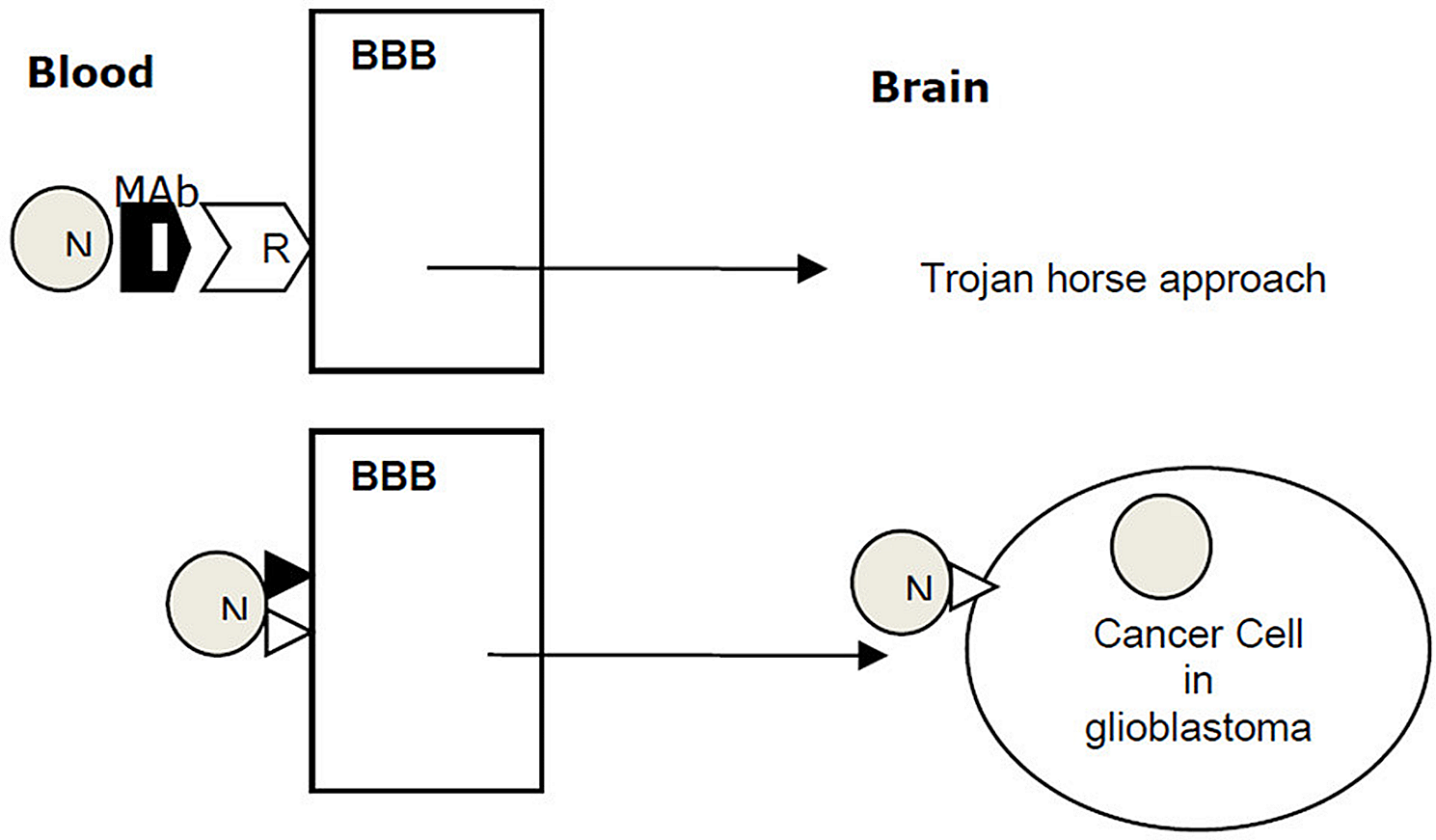
New approaches in GBM targeted therapy across the BBB. (Reprint from open access article [56]) (BBB: blood brain barrier, GBM: glioblastoma, N: nanoparticle, Mab: monoclonal antibody, R: receptor, ◮: Ligand)

Various methods have been demonstrated to enhance the transport of drugs across the BBB, with many of these techniques involving the disruption of the BBB. However, this disruption compromises the integrity of the cerebral microvasculature. One promising approach is the NP-based delivery of anticancer agents. Polymer and lipid NPs are commonly utilized as nanovehicles for delivering anticancer therapeutics. Figure 1 represents new approaches in GBM targeted therapy [56].

## New approaches in cancer therapy

Cancer is a dynamic ailment characterized by continuous evolution and outcomes of unpredictable nature [57]. In recent years, several important advances have been made in treatment approaches in clinical oncology with attention to the patient’s genetic/genomic profile, immunotherapy and more targeted therapy [54]. One of the new strategies in cancer therapy is the use of drug delivery carriers that are able to bypass cell barriers. Extracellular vesicles (EVs), which are a family of naturally occurring and cell-derived particles, can be used as drug delivery vectors because they are biocompatible and have a natural role in intercellular communication [57]. Due to their extremely small dimensions, exosomes can effectively traverse various tissue barriers without being engulfed by macrophages. This capability is not only attributed to their small size but also to the limited CD55 and CD59 expression, preventing opsonin and coagulation factor activation. Moreover, exosomes exploit various surface proteins to facilitate cellular internalization through endocytosis, making them highly efficient in drug delivery. The encapsulation of drugs within exosomes provides protection against circulating degrading enzymes, enhancing their potential for successful therapeutic delivery [58].

Starting from the mid-1980s, medical practitioners began to recognize the significance of targeted therapeutic approaches in cancer treatment, utilizing organic nanomaterials like liposomes [59]. By employing membrane fusogenic liposomes (MFLs), TPZ can be specifically delivered to distinct cellular compartments, such as the endoplasmic reticulum, and subsequently incorporated into newly formed exosomes released into the tumor microenvironment [58]. The use of glutathione, renowned for its antioxidant properties, as a targeting ligand involves coupling it to PEGylated liposomes, thereby enhancing their uptake into brain tissue through the glutathione transporter [60]. In an in vivo investigation using female athymic Friend leukemia virus B mice challenged with human glioblastoma cells (U87MG), it was demonstrated that DOX-loaded glutathione PEGylated liposomes (95 nm diameter; administered intravenously) increased the median survival time by 38.5% compared to mice treated with saline. This formulation not only improved the solubility and activity of DOX but also mitigated side effects, showcasing its potential in cancer treatment [61].

Nanoparticles have great potential for molecular targeting of cancer cells and drug delivery. This is extremely valuable in central nervous system (CNS) oncology, where the presence of BBB is a major obstacle in the drug delivery process [54]. Nanoparticles also can be used in combination with chemotherapy (CDT), photodynamic therapy (PDT), and sonodynamic therapy (SDT) [51]. It should be kept in mind that cancer cells in the early stages of growth are less likely to have mutations causing drug resistance [62]. In the initial priming stage, one of the strategies to fight cancer progression is to increase the delivery of the vaccine to the lymph nodes. Due to the size-dependent nature of lymphatic uptake, nanoparticle-encapsulated antigens are much more effective than unformulated vaccines, a concept which is now being more rigorously pursued by bioengineering-based strategies [63]. A characteristic of cancer cells is metabolic reprogramming, which helps these cells resist anti-cancer treatments. Glycogen metabolism is involved in the metabolic reprogramming of them under stress conditions such as hypoxia, glucose deprivation, or anticancer therapy. In the light of this, targeting of glycogen metabolic pathways has become a promising strategy for combination therapeutic approaches in cancer therapy [64].

### Nanotechnology

Ever since the approval of the first-generation nanotechnologies for clinical use, scientists have taken the liberty of navigating the tumor vasculature, since the hydrophilic coating of these nanomaterials renders them capable of penetrating into the tissue without getting opsonized and release their therapeutic content in a controlled manner. Second-generation nanoplatforms, on the other hand, are currently being appraised in clinical trials for combinatorial drug delivery purposes. Concurrently, development of a third generation of nanotechnological innovations have been initiated to deliver means for immune system modulation and self-recognition [65]. Nanosurgery in the field of targeted treatment is one of the new cure methods that can be used to remove residual microtumors or individual cancer cells after macroscopic surgery in organs. These residual microtumors cause tumor recurrence after surgery. In this method, several organic and inorganic nanoparticles are used for accurate detection and removal of microtumors [54]. Nanoparticles responsive to light represent powerful tools for nanosurgery and cancer treatment, displaying high effectiveness as agents for destroying cells. These nanoparticles can be precisely directed to specific cell types using appropriate recognition molecules [66]. In vivo experiments have successfully applied this technology to biological systems, with recent trials on mice demonstrating its effectiveness in achieving complete remission and the elimination of malignant tumors. These promising results are poised to progress into human clinical trials soon [67]. In a study conducted by Karabeber et al., a handheld Raman scanner was employed to evaluate the extent of resection in GBM, the most malignant form of brain cancer, within a genetically engineered mouse model. The handheld scanner accurately detected gold–silica surface-enhanced Raman scattering nanoparticles embedded in GBM, resulting in the complete resection of the tumor [68]. Further advancements in this strategy involve tailoring nanoparticle size for proximity to the operating room, overcoming blood-brain barrier challenges, and optimizing functionalization of nanoparticle conjugates to achieve maximum target concentrations [69].

#### The first, second and third-generation nanotechnological innovations

Nanovectors, whether hollow or solid, serve as nanoparticles with diverse applications in anticancer drug delivery, targeting moieties, and detection, thereby reducing toxic effects. These nanotechnological devices have garnered interest for their potential in cancer drug delivery and imaging [66, 70]. Nanovectors can be categorized into different generations [71]. The first generation non-specifically targets surface receptors on tumor cells [72]. An example is albumin-bound Paclitaxel used in breast cancer chemotherapy [73]. The second generation of nanoparticle technology focuses on active targeting, designed to identify and target specific biological molecules present on cancer cells. This approach incorporates high-affinity ligands and specific antigens on the surfaces of nanoparticles [74]. The ongoing development of the third generation involves a multi-stage strategy [75]. In the initial stage, biodegradable silicon microparticles with pores are designed to navigate the circulatory system and recognize endothelium specific to the disease. The subsequent stage comprises various nanoparticles loaded within the first-stage particles, released specifically toward the tumor mass. These nanoparticles, each smaller than 20 nm, can easily traverse interendothelial junctions and carry diverse payloads for both therapy and imaging, presenting a promising direction for future applications in cancer treatment [76].

### Nanomaterials; application of nanoparticles in cancer therapy

With a size of 1 to 100 nanometers, nanomaterials are an ever-expanding family of materials with unique electrical, magnetic, and optical features, that can be modulated for enhanced delivery and release of drugs into the tumor microenvironment. Despite an increasing number of investigations, the approval of nanomedicines has seen limited growth in recent years [40]. Organic NPs are known drug delivery systems with controlled release. The first attempts to synthesize organic polymeric nanostructures mainly involved lipid molecules as organic substrates or monomers. Lipids, especially phospholipid derivatives, can produce physical micronanostructures without any chemical intervention [77]. Consisting of metal ions with organic bonds, metal-organic frameworks (MOFs) are a class of molecular crystalline materials that are used for hierarchical integration of NPs and/or biomolecules into a single framework to functionalize them. As such, MOF-protected heterostructures ensure enhance the catalytic capacity of nanoparticles, without sacrificing the intracellular biological activity of biomolecules. These structures can be used in combination with photothermal treatment, chemotherapy, radiotherapy, immunotherapy and theranostics [78]. In addition to organometallic frameworks, covalent organic framework nanoparticles were also used for antitumor therapy. Porphyrin-based covalent organic framework NPs (COF-366 NPs) are an example of such structures that provide simultaneous PDT and PTT treatment with photoacoustic imaging (PA) monitoring, making the procedure simpler and It makes it easier. COF-366 nanoparticles attained a good phototherapy effect even in the case of sizeable tumors [79]. Among the advances of nanotechnology in cancer treatment is the development of nanomaterials that generate ROS, which may aggravate cell death by upregulating intratumoral oxidative stress. Various nanomaterials contribute to ROS production in tumor cells, and thus disrupting their redox balance, which leads to lipid peroxidation and oxidative damage to DNA and proteins [51].

Hyaluronic acid (HA) is the principal constituent of the extracellular matrix (ECM), which is known to be upregulated in initial stages of tumorigenesis. HA is incorporated into various types of nanomaterials, including micelles, polymersomes, hydrogels, and inorganic nanoparticle formulations, and HA-based nanomaterials play an important role in drug delivery systems [80]. HA is a prevalent glycosaminoglycan (GAG) found in the brain, where it forms a hydrogel-like mesh by interacting with other GAGs and proteoglycans [81]. HA exhibits exceptional physicochemical features, including high water-binding capacity, non-toxicity, biodegradability, cytocompatibility, and nonimmunogenicity [82]. The impressive biological characteristics of hyaluronic acid (HA) have generated considerable enthusiasm for the creation of nanomaterials based on HA, particularly for diverse biomedical purposes like drug delivery systems (DDS) and molecular imaging [80]. Numerous cancer cells, including those in GBM tumors, are known to overexpress HA-binding receptors like CD44, LYVE-1 receptors, and RHAMM [83]. Several studies have highlighted the overexpression of HA in GBM tumors and its impact on cancer progression [81]. The incorporation of an active moiety, such as HA, onto the surface of nanoparticles (NPs), facilitates active targeting, thereby enhancing cancer cell selectivity [84].

The rising 2D materials exhibit tremendous potential across various applications, spanning photoelectronics, water splitting, and energy storage. Leveraging their planar morphology, these 2D nanomaterials showcase distinctive physical, chemical, and mechanical properties, often uncommon in traditional bulk materials and their zero-dimensional (0D) or one-dimensional (1D) counterparts [85]. Due to the confinement of electrons in the 2D space, these nanomaterials typically exhibit intriguing electronic properties. Due to their very thin thickness, two-dimensional nanomaterials show unique mechanical, physical and chemical properties, which are very desirable for many applications, including disease diagnosis and treatment. Drug delivery, photodynamic therapy (PDT) and photothermal therapy (PTT) in cancer stand among the applications of these nanoparticles. Graphene and its derivatives are the first two-dimensional nanomaterials that were used to deliver anti-cancer drugs [86].

Metal sulfide nanomaterials (MeSNs) represent a novel class of nanomaterials known for their elevated biocompatibility and distinctive attributes in cancer therapy. These characteristics include Fenton catalysis, light conversion, radiation enhancement, and activation of the immune system. The unaltered MeSNs can effectively convert energy for both phototherapy and radiotherapy, giving them synergistic antitumor properties, a significant advantage over other nano-therapeutic agents. The effectiveness of MeSNs in treatment depends on intrinsic factors such as their accumulation within the tumor site. Despite their impressive anti-tumor effects, the utilization of MeSNs in life sciences is still in its early stages [87].

Utilizing a nanoprecipitation method enables the production of NPs using biodegradable and biocompatible polyester homopolymers like polylactic acid (PLA), polylactic-co-glycolic acid (PLGA), and polycaprolactone (PCL). These polymers have the capability to encapsulate or adsorb drug compounds. With suitable functionalization, they can improve the delivery of both hydrophobic and hydrophilic small drug molecules to designated target sites [88].

**Fig. 2:**
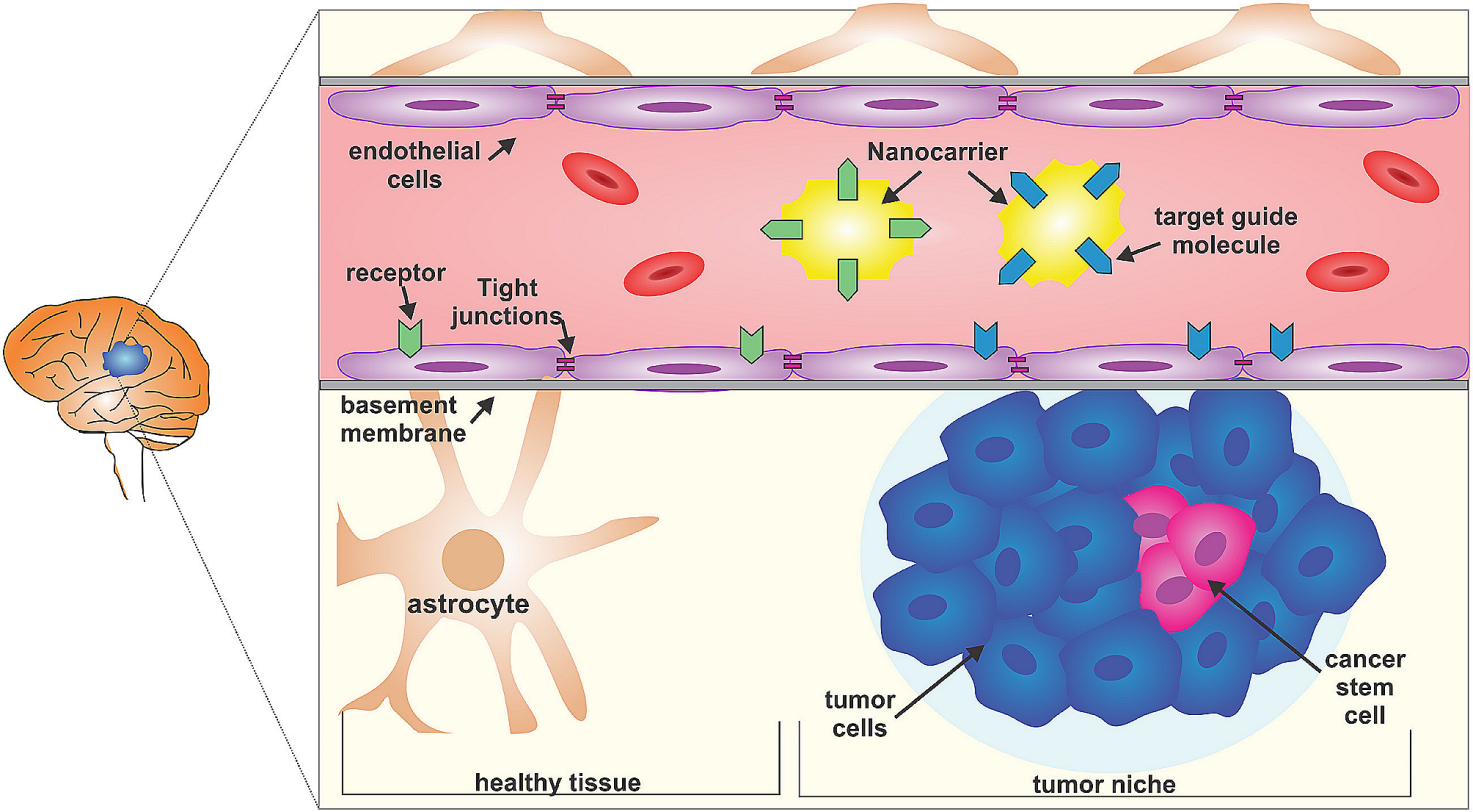
Different strategies in GBM therapy using nanotechnology. Reprint from open access article [89])

The BBB poses a challenge due to its selective permeability. Using nanocarriers, equipped with targeting molecules, offers a potential strategy to reach the glioma core. These molecules can bind to membrane receptors on both tumor-niche infiltrated BBB and healthy BBB, facilitating the transport of nanomedicines. Glioblastoma, with its heterogeneous cell populations, includes cancer stem cells responsible for treatment resistance. Figure 2 represents different strategies in GBM therapy using nanotechnology [89].

#### Drug delivery barriers to GBM

A persisting challenge in the brain cancer theranostics is the BBB, which consists of endothelial cells, astrocytes, and the basement membrane lying in between that together form a structural and functional barrier to protect the brain parenchyma from potentially hazardous compounds in the blood [56]. BBB limits cerebral drug delivery, which is of particular importance at the periphery of tumors, where tumor cells invade the neighboring intact tissue [56]. Small-sized nanoparticles may cross the BBB barrier and deliver drugs to the target site [90]. The advantage of neutral and anionic small-size nanoparticles (20–70 nm) is that they cause less neurotoxicity [91]. In contrast, metal nanoparticles (e.g., copper, silver and aluminum) may be more neurotoxic [54]. Following the change in the function and organization of the BBB due to the increase in the severity of GBM malignancy, the blood brain tumor barrier (BBTB) is formed. BBTB limits the penetration of drug delivery systems [92]. Proper release of drugs is also necessary for effective treatment. Following the aggressive invasion of GBM, the migration of cancer cells to the neighboring tissues of the brain occurs. After tumor surgery, the migrating cancer cells may become recurrent GBM adjacent to the original tumor area [92]. Most current topical delivery systems that bypass the BBB cover only a small area near the delivery site, a shortcoming that needs to be addressed [93]. Also, low drug release may bring about high toxicity at local delivery sites [94]. Another major barrier against the efficacy of anticancer treatment is MDR [95]. Inherent resistance to chemotherapy drugs exists in certain cancer cells, while others acquire this trait through mutations during the carcinogenesis process [96]. Multi-Drug Resistance (MDR) arises due to the upregulation of the ATP-binding cassette (ABC) transmembrane transporter superfamily. Among the frequently overexpressed members of ATP-ABC, MDR-associated protein-1 (MRP1/ABCC1), breast cancer resistance proteins (ABCG2), and P-glycoprotein (P-gp/ABCB1) are notable. The heightened expression of these proteins reduces the effective intracellular concentration of chemotherapeutic agents in an ATP-dependent manner. Another mechanism leading to MDR involves the redistribution of drug molecules away from the target site, facilitated by non-ABC drug transporters like lung resistance protein (LRP; major vault protein). Additionally, the cytotoxic effects of chemotherapeutic drugs directed at tumor cells can be countered by detoxification mechanisms. Noteworthy drug-metabolizing enzymes that contribute to drug inactivation and exhibit increased expression in malignant cells include Glutathione-S-transferases, cytochrome P4503A, and aldehyde dehydrogenase-related phase II [97].

#### Drug delivery using targeted therapy

Poor penetrability often compromises the efficacy of treatment [54]. In contrast to this, though, in targeted drug delivery the therapeutic agent accumulates at the target site via the circulation. Based on the mechanism of delivery, targeted therapy can be classified into two major categories: (1) Passive targeting: in which the therapeutic particles are intercepted as a result of physiological phenomena such as enhanced permeability and retention (EPR) effect at tumor tissue; and (2) Active targeting: in which the therapeutic agent is modified by a specific ligand, the receptor of which is amply expressed at the target site. Combination of both classes, as in modification of particles with certain morphological features, would result in better delivery compared to either class alone. As such, application of passive targeting strategy, in the case of GBM that is inherently associated with BBB-induced inaccessibility, would render the treatment ineffective [98]. Owing to a feature termed ‘controlled release reservoir’ nanoparticles have been shown to be quite effective at releasing therapeutic agents within a good proximity of the target site. However, certain criteria must be met before clinical adoption of these nanoparticles such as biocompatibility, since a primary goal in targeted therapy is to avoid the adverse events caused by conventional therapy in the first place [99].

Jallouli and colleagues investigated the permeability of 60 nm porous nanoparticles with maltodextrin backbones, comparing cationic and neutral variants, in an in vitro BBB model known for its correlation with in vivo observations. Neutral NPs were observed to traverse endothelial cells through caveolae-dependent transcytosis, potentially mediated by glucose transporter (GLUT-1) and/or lectins. Both cationic and neutral NPs successfully traversed the BBB model via lectin-dependent transcytosis, although the efficiency of cationic NP transcytosis was lower [100].

These results imply that surface charge could influence binding to and passage through endothelial cells, making both cationic and neutral porous NPs potential candidates for brain drug delivery. A common method to modify NP surfaces is PEGylation, which involves the conjugation of PEG. PEGylation has been proven to reduce opsonization, resulting in decreased uptake by the reticuloendothelial system (RES) and prolonged circulating half-lives of PEGylated NPs [101]. In a study conducted by Zhao et al., a GBM mouse model was employed to demonstrate the safety and efficacy of PEGylated PAMAM dendrimer NPs when conjugated with the CREKA peptide. These PEGylated NPs exhibited prolonged in vivo circulation compared to uncoated NPs, alleviated the inherent toxicity of PAMAM, and achieved deep penetration into GBM tissue [102]. Gref et al. designed sterically stabilized nanospheres using amphiphilic diblock or multiblock copolymers. These nanospheres featured a hydrophilic PEG coating and a biodegradable core encapsulating various drugs. Hydrophobic drugs, such as lidocaine, were successfully entrapped up to 45 wt%, and the release kinetics were influenced by the physico-chemical characteristics of the polymer. The PEG-coated particles demonstrated a significant reduction in plasma protein adsorption compared to non-coated ones, with varying protein amounts over time. The nanospheres displayed prolonged blood circulation times and reduced liver accumulation, depending on the molecular weight and surface density of the PEG coating. Furthermore, they could be freeze-dried and redispersed in aqueous solutions, showcasing good shelf stability. This approach introduces the possibility of tailoring “optimal” polymers for specific therapeutic applications [103].

A multitude of in vivo investigations, encompassing both rodents in good health and a mouse model with breast cancer, provide substantial evidence affirming that PEGylated nanoparticles efficiently prolong the circulation of nanocarriers in the bloodstream and improve the stability of NP formulations when contrasted with their uncoated counterparts [104].

Moreover, bioactive compounds present in seeds, vegetables, and fruits possess antioxidant, anti-inflammatory, and anticancer attributes that could enhance the well-being of cancer survivors during chemotherapy or other treatments. The integration of these compounds into nanocarrier-based drug delivery systems for addressing GBMs presents a potential therapeutic approach for this type of tumor. This strategy aims to enhance targeting precision, elevate bioavailability, and minimize side effects by improving drug internalization into cells. Simultaneously, it reduces the likelihood of off-target organ accumulation [105].

## Inorganic nanoparticles

Inherent features of inorganic nanoparticles encompass customizable morphology and nanostructure, straightforward functionalization, commendable physiological stability, and distinctive physicochemical properties like optical, electrical, acoustic, and magnetic attributes, setting them apart from conventional organic or polymer-based counterparts [106].

### Magnetic nanoparticles (MNPs)

In the past ten years, advancements in research have substantially enhanced the theranostic capabilities of MNPs in cancer nanomedicine. The latest MNPs offer several benefits, including broader operating temperature ranges, reduced sizes, lower toxicity, simpler preparation methods, and decreased production costs. Due to their distinct and superior physical and chemical characteristics, MNPs show significant promise in various medical applications. Specifically, they can be utilized as probes in medical imaging and as carriers in targeted drug delivery systems [107].

Inorganic NP-based systems, specifically magnetic nanoparticles (MNPs), manifest in two primary types: (i) IO cores coated with a polymer surface and (ii) NPs with embedded crystals of IO [33]. These nanoparticles are usually composed of pure metals (Fe, Co, Ni, and some rare earth metals) or a mixture of metals and polymers, offering elevated magnetic moments and surface-area-to-volume ratios that make them attractive for hyperthermia therapy in cancer and targeted drug delivery. Additionally, they can act as contrast agents for magnetic resonance imaging (MRI) and enhance the sensitivity of biosensors and diagnostic tools [108]. Typically, MNPs with a size smaller than 50 nm exhibit superparamagnetism, mainly consisting of superparamagnetic iron oxide nanoparticles (SPIONs) [107].

Magnetic nanomaterials possess several characteristics: They display extensive specific surface areas [36], and have the ability to transport a variety of small molecules, proteins, RNA, and more. The magnetic properties of nanometal particles facilitate their enrichment, separation, movement, and precise positioning [37]; Moreover, MNPs exhibit a magnetocaloric effect in a high-frequency magnetic field, potentially leading to the indirect eradication of tumor cells [38]. At present, magnetic nanoparticles (MNPs) are extensively utilized in the field of medicine, encompassing applications such as drug delivery [107]. MNPs are extensively employed in various cancer theranostics including magnetic hyperthermia and resonance imaging, PDT, and PTT [109]. Coating the surface of MNPs with other materials serves the purpose of enhancing their colloidal stability, allowing for the attachment of therapeutic cargoes, and regulating the pharmacokinetics and pharmacodynamics of MNPs [109]. Traditional approaches to magnetic nanoparticle (MNP) synthesis include the co-precipitation of salts with stabilizing polymers, hydrothermal or thermal solution techniques, sonochemistry, reverse microemulsion, and thermal decomposition. Recently developed synthesis methods encompass microfluidic and biogenic approaches [109].

As a drug delivery system, MNPs can be loaded with anticancer therapeutic agents such as curcumin, TMZ, and PTX, resulting in the suppression of GBM tumor cell proliferation [110]. In their study, Rezaie et al. (2018) coated their magnetic nanoparticles with poly(caprolactone)-poly(ethylene glycol) (PCL-PEG) as a carrier of 5-iodo 2’deoxyuridine (IUdR), which was later administrated to U87MG glioblastoma cell cultures in the presence of hyperthermia. Their observations confirmed a decrease in the number of colonies of spheroid glioblastoma cells treated with IUdR or nanoparticle-encapsulated IUdR, which can be said to be magnetic nanoparticles coated with PCL- In addition to being an effective means of transporting IUdR into cells, PEG can act as a radiation sensitizer and heat sensitizer in the treatment of glioblastoma cell lines [111].

#### Iron oxide NPs (IOMNPs)

A mostly commonly lab-synthesized nanoscale particle [112], magnetic iron oxide (either γ-Fe_2_O_3_ or Fe_3_O_4_) nanoparticles are the most extensively used type of NPs in the field of cancer theranostics, as they are both reactive to magnetic currents and well-tolerated by the patients [109]. As their therapeutic effects are temperature-dependent [113], IOMNPs have been used at different temperatures in several investigations [114]. Size and surface functionality play an important role in the pharmaceutical applications of IOMNPs [115]. Nanoparticles with a size larger than 200 nm are readily filtered by the reticuloendothelial system (RES). Excessively small particles (< 8 nm), on the other hand, are easily eliminated from the body through excretion in the urine [116] and their blood circulation time is reduced. Particles with a size of 10–40 nm (including very small IOMNPs) persist the longest in blood circulation [114], and can be stabilized at the target size by applying an external magnetic field, which in turn mitigates the required dose and potential adverse effects. As the therapeutic success highly depends on the composition of the outer coating, polymer layers, capsules, particles or vesicles have been proposed for use as the outer layer. Surface modifications of these particles are carried out using organic polymers and metals or inorganic oxides [117].

With IOMNPs, higher levels of efficacy are attained with homogeneous dispersion of the NPs in an aqueous media along with the addition of functional groups, which can be used for attachment of targeting units [116]. Considering the reactive area of IOMNPs and their capability to cross biological barriers, they stand among the NPs of choice for clinical application [116]. In this context, anticancer agents such as doxorubicin, docetaxel, 5-fluorouracil, gemcitabine and methotrexate can be encapsulated within IOMNPs [117, 118]. Studies show that IONPs are able to stimulate immune effects mediated by T cells against tumors. Once they are accumulated at the tumor site, IOMNPs can generate heat under an external alternating magnetic field and kill cancer cells, so they enhance immune function in the Tumor microenvironment by releasing pro-inflammatory cytokines [119]. IOMNPs can activate NADPH oxidases, induce the formation of reactive oxygen species and promote an imbalance in redox homeostasis, which renders them a highly effective tool for killing of malignant cells [120].

### Gold nanoparticles (GNPs or AuNPs)

Gold nanoparticles (GNPs) have been used as tumor-specific drug carriers, imaging agents, radiosensitizers, and anti-angiogenic agents due to their easily controllable and modified shape, size, and surface chemistry, as well as biocompatibility and less cytotoxicity [121]. EPR is one of the factors that facilitate their penetration inside tumors [122]. In vitro experiments show that GNPs exert their cytotoxic effects in cells through induction of oxidative stress [123]. Cell apoptosis through the generation of oxidative stress is an important mechanism of GNP toxicity [124]. ROS may disrupt the balance between oxidant and antioxidant cellular processes [123, 124]. According to recent studies, the size-dependent cytotoxicity of gold nanoparticles is enhanced the deeper they penetrate into the target tissue [123]. In their study, Chen et al. used BSA-coated gold nanoparticles (BSA AuNPs) as radiation sensitizers, suggesting that 18 nm BSA-AuNPs may repress colony formation and induce DNA double-strand breaks (DSBs) in glioblastoma cells, compared to radiotherapy alone. Damage to cell membrane and mitochondria, contribute to ROS formation and cell cycle arrest [125]. U2-AuNP (conjugating aptamer U2 with gold nanoparticle) is a nanoparticle based on gold particles that was synthesized by Peng et al. and its effect on U2-AuNP cell line was investigated. Also, the antitumor effects of this nanoparticle were investigated in the body of mice with glioblastoma, the findings of which indicated that U2-AuNP inhibits the proliferation and invasion of U87-EGFRvIII cell lines and EGFR-related pathway, preventing DNA damage repair in GBM cells [126].

### Carbon nanotubes (CNTs)

**Fig. 3:**
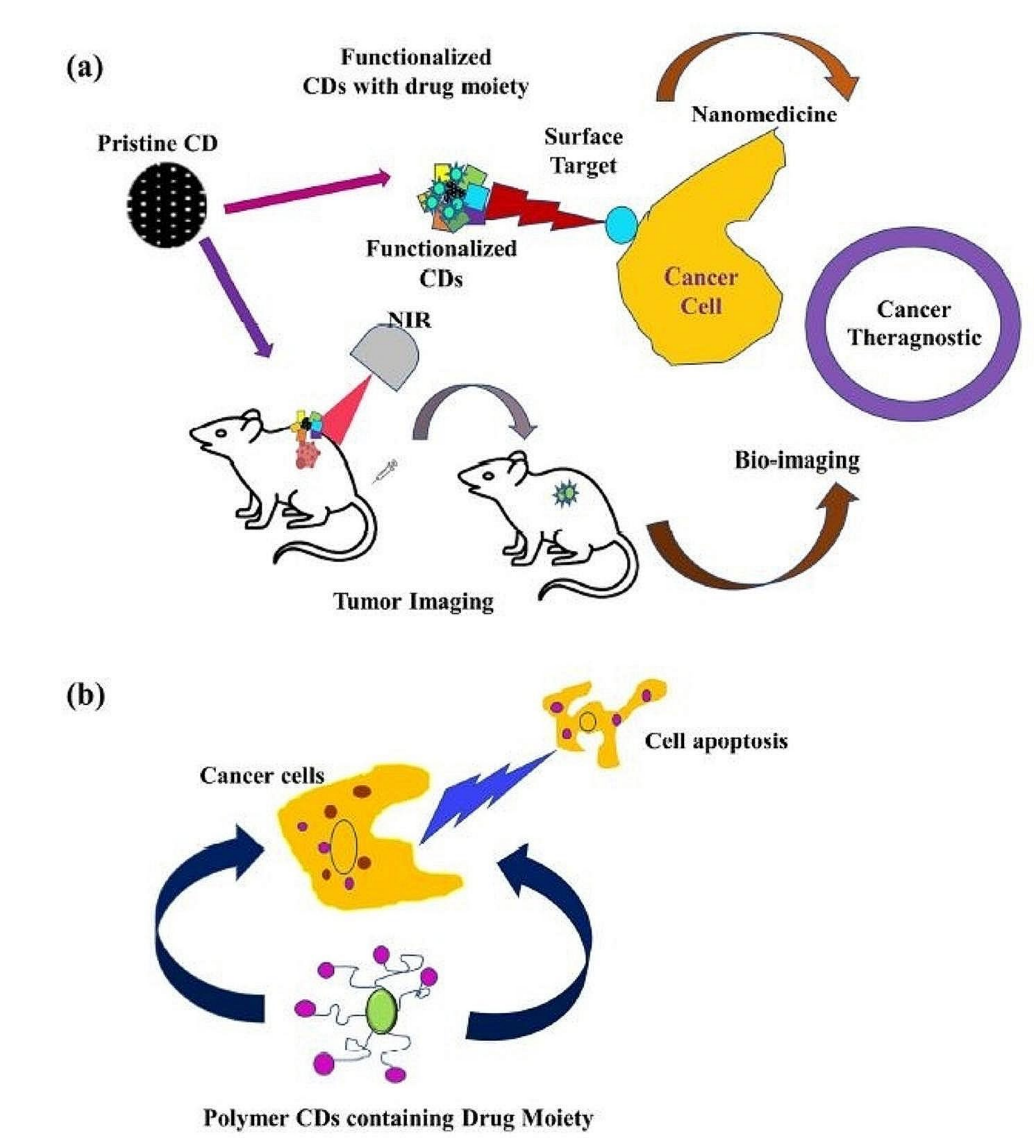
Utilizing carbon dots for cancer theranostic near-infrared (NIR) bio-imaging (**a**) and studying apoptosis in the context of cancer cell death. (Reprint from open access article [127])

Recently, carbon dots (CDs) have been extensively explored for their various properties [127]. Carbon nanomaterials (CNMs) such as graphene, and carbon nanotubes and quantum dots, are another category of nanomaterials with high capability of targeting cancer cells [128]. Carbon nanotubes (CNTs) are generated from cylindrical graphite sheets [129], and are highly stable, biocompatible, non-immunogenic particles with particularly high value in targeted drug delivery [130]. ROS production is one of the main mechanisms of antitumor effects of carbon nanoparticles. Also, the large surface area of carbon nanoparticles absorbs other chemical substances, which after biotransformation, can be oxidized to redox active quinones. The antitumor effects of CNPs are attributed to downregulation of matrix metalloproteinases (MMPs) which inhibits tumor metastasis, and enhancement of antitumor immunity secondary to ROS generation and activation of toll-like receptors (TLRs) in phagocytes [120]. The use of CNTs for targeted tumor heating is one of the studied methods in the treatment of glioblastoma. These nanoparticles can convert near-infrared light into heat, thus, heating up tumor cells only to destroy them [131]. Carmustine is an anticancer drug carried by Gliadel, with notable side effects when used traditionally. Nitrogen-doped CN sponges (N-CNSs) can be loaded with large amounts of hydrophobic drugs and reduce the amount of carriers. In a study, these nanomaterials were evaluated as carriers of carmustine using a malignant glioma cell line. The results showed that N-CNSs, at concentrations below 40 μg/mL, did not exhibit significant cytotoxic effects. Carmustine-loaded N-CNSs were able to continuously release carmustine up to 72 h from initial administration, with adverse effects comparable to that of carmustine alone [132]. Figure 3 illustrates CDs as theranostic agents in cancer [127].

### Quantum dots (QDs)

Quantum dots (QDs), distinguished by their nanoscale dimensions and unique optical and electronic properties, are proving to be a revolutionary tool in the treatment of glioblastoma, an exceptionally aggressive form of brain cancer. In the pursuit of more effective therapeutic strategies, graphene quantum dots (GQDs) have emerged as promising candidates due to their biocompatibility and distinctive photophysical attributes [133]. A seminal study conducted by Perini et al. in 2023 delved into the multifaceted potential of GQDs in combatting glioblastoma. These nanoparticles exhibited a remarkable ability to traverse the formidable blood-brain barrier, a critical challenge in brain cancer treatment. In a 3D spheroid model of glioblastoma, surface-functionalized GQDs not only enhanced membrane fluidity and intracellular uptake but also synergized with antitumor drugs like doxorubicin and temozolomide at subtherapeutic doses. The study unveiled a novel therapeutic strategy termed photothermal therapy (PTT), wherein GQDs absorbed and converted near-infrared light into heat, enhancing membrane permeability and amplifying the effects of chemotherapy. The combined PTT and chemotherapy approach significantly reduced tumor growth and viability, demonstrating the potential of GQDs in mitigating side effects and directing the immune response for improved patient quality of life [134].

The groundbreaking findings were complemented by the implementation of INSIDIA 2.0, a user-friendly image analysis software introduced by Perini et al. in 2022. This software facilitated high-throughput and high-content quantitative analysis of in vitro 3D cancer cell spheroids, offering a rapid and efficient means of assessing the effects of GQD photothermal therapy on glioblastoma and pancreatic cancer spheroids. The advanced parametrization of spheroid morphological changes provided crucial insights, allowing researchers to quantify cell death in a non-invasive, fast, and high-throughput fashion. The software’s ability to analyze the impact of GQD photothermal therapy on U87 glioblastoma spheroids revealed a decrease in the spheroid area accompanied by the generation of a high uniform density spheroid core, emphasizing the potential of GQDs in inducing targeted cell death [135].

Expanding the horizon of quantum dot applications, Li et al. (2022) explored the use of Nd3 + ion-coordinated black phosphorus quantum dots (BPNd) for targeted therapy against glioblastoma. BPNd exhibited superior performances in second near-infrared (NIR-II) fluorescence imaging and X-ray-induced photodynamic therapy. The study highlighted the optoelectronic switching effects between BPNd and Nd3 + ion, enabling precise monitoring of glioblastoma growth through intracranial NIR-II fluorescence imaging and inhibiting its progression through targeted X-ray-induced photodynamic chemotherapy. The ultrasmall size of BPNd, coupled with its efficient cargo loading capacity, facilitated its crossing of the BBB, providing a promising avenue for precise and effective treatment strategies against glioblastoma [136].

The cumulative evidence from these studies underscores the transformative potential of quantum dots in advancing targeted and effective therapies against glioblastoma. Whether through GQDs’ multifaceted applications in PTT and chemotherapy or BPNd’s innovative use in NIR-II fluorescence imaging and X-ray-induced photodynamic therapy, quantum dots hold immense promise in reshaping the landscape of glioblastoma treatment. These advancements not only improve therapeutic outcomes but also pave the way for reduced side effects and enhanced patient well-being in the challenging realm of glioblastoma management [137].

## Glioblastoma therapy

### Monotherapy

Effectively delivering therapeutic agents to the tumor site while minimizing impact on normal tissues is a significant challenge in the realm of cancer treatment. The utilization of nanocarriers in conjunction with various treatment modalities like chemotherapy agents, PTT, PDT, and RT-RDT holds the potential to enhance the efficacy of these approaches [138]. The application of specific nanoparticles (NPs) in monotherapy for glioblastoma is summarized in Table 1. Eugenio et al.‘s investigations into silver/silver chloride nanoparticles (Ag/AgCl-NPs) on GBM02 cells revealed a notable reduction in tumor cell growth. Notably, the inhibitory effect at higher concentrations surpassed the impact of Ag/AgCl-NPs and temozolomide in diminishing cell growth [139]. Inhibition of histone deacetylases (HDACs) can induce cell cycle arrest, enhance cell differentiation, and trigger apoptosis. Kesinostat, an HDAC inhibitor, faces limitations in its use as monotherapy against solid tumors due to poor delivery. However, positive outcomes were observed when Kesinostat was encapsulated in poly (D, L-lactide)-b-methoxy poly (ethylene glycol) nanoparticles (NPs), leading to increased survival rates in laboratory rodents [140]. Exploring different light sources in photodynamic therapy, Davanzo et al. (2017) employed human serum albumin nanoparticles containing chloro-aluminum phthalocyanine (AlClPc) on U87MG cells. Three distinct light sources with doses of 200, 500, and 700 mJ/cm2 were utilized, and the evaluation of cell death pathways through flow cytometry revealed apoptosis as the predominant mode of cell death in all cases [141].

**Table 1:** Application of some nanoparticles in glioblastoma monotherapy

Monotherapy						
Treatment modality	Nanocarriers	Cancer cell types	Loading efficiency	Release rate	Animal models	Refs.
None	PLA-PEG	GL261-LucNeo	9%	50%(4 h,37 °C)	Mice bearing GL261-LucNeo tumors	[140]
Radiation therapy	αPD-L1-LNP	TAMCs	-	-	GL261 glioma-bearing mice	[142]
Irradiation	PVP	Rat C6 glioma cells	-	-	Rats bearing glioma	[143]
chemiexcited PDT	M@HLPC	U251	-	-	Mice bearing glioma	[5]
None	Polymeric Nanoparticles	U87MG	-	pH:7.4 37 ◦C 73.05% of TMZ and 91.81% of Gen	-	[144]
Magnetic hyperthermia	M-PLL IONP	U87-Luc	-	-	Mice bearing glioma	[145]
near-infrared fluorescence guided	biodegradable fluorescent mini nano imaging agent (NIA)	U87MG	-	-	xenogeneic mouse model	[146]
Chemotherapy	Carbon-dots	SJGBM2, CHLA266, CHLA 200 U87	-	-	-	[147]
Photothermal Therapy	Carbon Nanodots	U87	-	-	mice bearing U87 GBM tumors	[148]
Photothermal Therapy	Silica-Coated Gold Nanorods	N2a	-	-	N2a glioma-bearing mice	[149]
None	Polymeric Micelle	U87MG HUVEC	-	-	mice bearing U87 GBM	[150]
None	Luteolin	GL261	-	12 h 79.2%	mice bearing GL261 GBM tumor	[151]
None	MPEG-PCL	C6 and U87	-	PH7.4 37 °C. 10 h, 46%	Mice bearing gliomas	[152]

### Combination therapy

Combinational treatments, as in combined nanocarriers, are associated with relatively higher therapeutic yield as a result of synergistic effects and the reduced required dose of each carrier. Recently, numerous types of nanoparticle-based therapeutic combinations such as photodynamic-immunotherapy or radioimmunotherapy and hyperthermo-chemotherapy, have been indicated for cancer therapy [138]. The delivery system or platform is the pillar of combinational therapeutic approaches, and should be selected in a way to facilitate loading and delivery of the multiple therapeutic agents [153]. According to the study of Hua et al., the use of 10 nm AgNPs in combination with IR treatment with MHT shows both radiosensitivity and thermal sensitivity on U251 glioma cells. Also, after RT, MHT and RT combined with MHT, AgNPs can significantly prevent the proliferation of cancer cells [154]. Application of some NPs in glioblastoma combination are summarized in Table 2.

**Table 2:** Application of some NPs in glioblastoma combination

Combination therapy						
Treatment modality	Nanocarriers	Cancer cell types	Loading efficiency	Release rate	Animal models	Refs.
Hyperthermia-chemotherapy	Fe(Salen)	U251	-	-	U251 bearing Mice	[155]
Magnetite chemo-hyperthermia	Magnetite nanoparticles functionalized with folic acid ligand	C6	65.6%	10 min pH 7.4 43 °C 44.4%	-	[156]
chemo-photothermal therapy	PLGA functionalized with angiopep-2 peptide	U87MG	78.6%	-	Tumor bearing mice	[157]
Chemo-immunotherapyc	MSN-SS-CD-iRGD&1MT nanoparticle	GL261-luc	-	-	GL261-luc bearing C57BL/6 mice	[158]
Magnetothermal chemotherapy	Fe-TSL	U87 cells and U251 cells	19.75%	80% in 10 min at 42 °C	-	[159]
Hyperthermia and chemotherapy	lipid-based magnetic nanovectors	U-87 MG	4.1%	pH 7.4 7 day 65.8%	-	[160]
Gene therapy hyperthermia	Cationic liposomes	U251-S	-	-	Tumor bearing mice	[161]
Chemotherapy and hyperthermia	Liposomes	C6	-	80% after 10 min, 37 °C	C6 bearing rats	[162]
Photodynamic Therapy and Chemotherapy	Cu2-xSe nanoparticles	U87	-	72 h, 11% at pH = 7.4	mice bearing orthotopic malignant glioblastoma	[163]
Ultrasound, Chemotherapy	lipid-polymer	T98G U251 U87 MG	2.5%	pH 4.5, 48 h, 12.5%	-	[164]
Radiation- gene therapy-immunotherapy	solid lipid nanoparticle (SLN) functionalized with cyclic peptide iRGD	U87 GL261	-	-	Glioblastoma bearing mouse	[165]
Radiotherapy and Immunotherapy	AuNPs	G261	-	-	tumor-bearing C57BL/6 mice G261 tumor-bearing mice	[166]

## Challenges of nanotechnology

Among the advantages of nanotechnology can be mentioned easy functionalization, increased sensitivity, and adjustable features [167]. One of the advantages of nanotechnology in medicine and cancer treatment is improving immunity. Due to their small size, nanoparticles easily create an immune response after administration inside the body. In the field of cancer immunotherapy, nanoparticles are used to deliver tumor antigens to APCs to enhance the immune response [167]. Also nanomaterials delivering antitumor agents to both primary tumors and the distant metastases [168]. On the other hand, there are also unwanted effects of using nanotechnology in oncology [169]. Although nanoparticles are widely used in cancer treatment, a major concern is the possible carcinogenicity of nanoparticles. Exposure to nanomaterials may cause genetic aberrations. This has been confirmed in research done in laboratory cell culture in the report of Zhang et al [157, 169]. Also, Singh et al. pointed out that several nanomaterials may cause nucleic acid abnormalities, and this is the underlying mechanism of nanogenology [169, 170].

Another important issue in the use of nanoparticles is to confirm their safety [171]. Nanoparticles can enter the human body through breathing. Shen et al. concluded that nanoparticles can be absorbed by the endocytosis process and can cause more damage to genes directly or indirectly. These damages can disrupt the physiological course of the cell cycle and lead to genome instability, which may eventually cause gene mutations or chromosome aberrations [172]. Another important issue is compliance with ethical principles in clinical studies. Those participating in clinical trials should receive thorough information about nanoexperiments in oncology. Concealing or incomplete data during testing of new nanomedicines in oncology is considered unethical and is a direct violation of the principle of prior informed consent [169].

## Translation of biomaterials to the clinic; clinical trials

Table 3 represents related clinical trials in GBM therapy through nanotechnology.

**Table 3:** Related clinical trials in GBM therapy through nanotechnology

Study ID	Title	Studied subjects	Condition	Intervention	Aim	Status
NCT04899908	Stereotactic Brain-directed Radiation With or Without Aguix Gadolinium-Based Nanoparticles in Brain Metastases	112 participants	• Brain Cancer • Brain Metastases • Melanoma Lung Cancer BC HER2-positive Breast Cancer CRC Gastrointestinal Cancer SRSSRTWhole Brain RadiationStereotactic RadiationAGuIX NPs Cystic Brain Tumor	• Radiation: Stereotactic Radiation • Drug: AGuIX gadolinium-based NPs • Other: Placebo	Treatment	Recruiting, 2021–2021, Phase 2
NCT04094077	Evaluating AGuIX® Nanoparticles in Combination With Stereotactic Radiation for Brain Metastases	1 participants	• Brain Metastases	• Drug: AGuIX	Treatment	Terminated 2019–2021 Phase 2
NCT02820454	Radio sensitization of Multiple Brain Metastases Using AGuIX Gadolinium Based Nanoparticles	15 participants	• Brain Metastases	• Drug: AGuIX • Radiation: whole brain radiation therapy	Treatment	Completed 2016–2019 Phase 1
NCT04881032	AGuIX Nanoparticles With Radiotherapy Plus Concomitant Temozolomide in the Treatment of Newly Diagnosed Glioblastoma	66 participants	• Glioblastoma	• rug: Polysiloxane Gd-Chelates based nanoparticles (AGuIX) • Radiation: radiotherapy • Drug: Temozolomide	Treatment	Recruiting 2021–2022 Phase 1 Phase 2

## Conclusion and future perspective

One of the important challenges in the field of cancer treatment is to reach the tumor site without affecting the normal tissues. Several studies have been conducted in this field, and the use of nanoparticles as a modern method has several advantages over traditional treatments. Multifunctionality, effective drug transport, and controlled release of drug delivery are among these features. The used nanosystems can be combined with different ligands. We mentioned that in malignant glioma, the BBB is the most important barrier for drugs to enter the targeted brain delivery system. Also, P-glycoprotein, using the energy released by the hydrolysis of adenosine triphosphate (ATP), pumps the used drugs out of the cell and reduces the effective concentration of the drug. Drugs used in the treatment of glioblastoma can be incorporated into NPs, and functionalized with various ligands to enable crossing and targeting the BBB. In addition to proper delivery, drug stability increases and unwanted side effects are reduced to some extent. Nanocarriers can be made from different materials such as organic materials, minerals, various metals and polymers. Also, biological materials such as protein and lipids have been used in this field, which have better efficiency in the production of translational nanotherapeutics. Temozolomide, paclitaxel, docetaxel, cisplatin, doxorubicin, curcumin, and nucleic acids are several anticancer drugs that have been delivered to the brain through nanosystems. The possible carcinogenicity of nanoparticles, and gene damage are among the main problems in the field of nanoparticle treatment that require further studies.

## Data Availability

No datasets were generated or analysed during the current study.
